# Treatment adherence of a case managed program for patients with severe schizophrenia compared to standard care in mental health units.

**DOI:** 10.1192/j.eurpsy.2023.2312

**Published:** 2023-07-19

**Authors:** S. Díaz-Fernández, J. J. Fernández-Miranda, F. López-Muñoz

**Affiliations:** 1 AGC Salud Mental V- HUCAB, Servicio de Salud del Principado de Asturias-SESPA, Gijón; 2 Health Sciencies, Universidad Camilo J. Cela, Madrid, Spain

## Abstract

**Introduction:**

Although some studies have reported that case management (CM), when is compared with standard care, reduces the loss of contact with health services, the debate continues about its superiority over other treatment models.

**Objectives:**

To assess treatment adherence and reasons for treatment discontinuation, and the impact of the type of APs administration on it, for a group of patients with schizophrenia treated in a CMP or receiving standard treatment in mental health units (MHUs).

**Methods:**

An observational, longitudinal study (ten-year follow-up) was conducted on 688 patients with severe schizophrenia (CGI-S ≥ 5). All the causes of the end of treatment were recorded, together with the AP medication prescribed and kind of regimes.

**Results:**

43.6% of the patients had discontinued treatment in MHUs and only 12.1% on the CMP (p < 0.0001). 27.6% of patients in MHUs were on long-acting injectables (LAIs), and 57.6 on the CMP (p < 0.001). Treatment discontinuation was closely linked to be on OAPs medication in both cases (p < 0.001).

**Table 1.** Treatment discontinuation, hospital admissions and suicide attempts [N(%)]

***N= 688***	**MHU (N=344)**	**CMP (N= 344)**	***P value***	
***Treatment discontinuation***	290 (84.3)	42 (12.2)	<0.00001	
	***OAP***	***LAI***	***OAP***	***LAI***
***Treatment discontinuation***	180(52.3)	90(26.2)^a^	34(9.9)	8(2.3)^b^
***Hospital admissions***	260 (75.6)	80 (23.5)	<0.001	
	***OAP***	***LAI***	***OAP***	***LAI***
***Hospital admissions***	180 (52.3)	80 (23.5)^a^	65 (18.9)	15 (4.4)^b^
***Suicide attempts***	134 (38.9)	26 (7.7)	<0.0001	
	***OAP***	***LAI***	***OAP***	***LAI***
***Suicide attempts***	160(46.5)	74(21.5)^a^	18(5.2)	8(2.3)^b^

*^a^: p<0.01 ^b^: p<0.001 N: number of patients %: percentage of patients*

*MHU: mental health unit CMP: case managed programme*

*AP: antipsychotic FGA, SGA: first, second generation antipsychotic*

*OAP: oral antipsychotic LAI: long-acting injectable antipsychotic*

**Conclusions:**

Our findings show how specific strategies as programs with an integrated treatment and case-managed approach, increase adherence. Moreover, treating with LAI APs clearly contributes to the achievement of these results. The widespread implementation of comprehensive community programs with case management, and the use of LAI-APs, should be an effective choice for people with schizophrenia and clinical severity and impairment, and at high risk of treatment discontinuation.

**Disclosure of Interest:**

None Declared

